# Four Women Who Refused Oophorectomy, and Their Subsequent Histories

**Published:** 1893-07

**Authors:** H. McHatton

**Affiliations:** Macon, Ga.


### FOUR WOMEN WHO REFUSED OÖPHORECTOMY, AND THEIR SUBSEQUENT HISTORIES.

BY H. McHATTON, M. D.,
MACON, GA.

CASE 1.—Widow, thirty-five years of age, mother of three children, has been under my care for the past seven years. Has an anteversion and painful menstruation; uterine condition normal in other respects. During my time of attention she has never been in bed a day on account of her menses. Has all the care of her family, which she has supported for years, which has involved a great deal of labor and mental solicitude. About eighteen months ago she was advised oöphorectomy as the only means to make her future bearable. The operation being insisted upon, she absolutely refused. She has had no professional treatment since, and tells me to-day that she is perfectly well, which assertion is fully sustained by her mental and physical condition, as well as by the amount of work that she is doing.

CASE 2.—Married two years, age twenty-two. One child nearly a year old. Gives a history of painful menstruation with the usual train of nervous and hysterical symptoms previous to her pregnancy. Just before her marriage, after due consultation, oöphorectomy was advised, insisted upon and refused. She is to-day the picture of a healthy young matron, and assures me that she is not even aware of the existence of her womb. I have never examined this case.

CASE 3.—Married, thirty-one years of age, mother of two children. Was one of the worst cases of dysmenorrhœa that I ever saw. In bed about ten days of each month. All varieties of hysterical symptoms, pronounced hystero-epilepsy, nervous tone never being regained during intra-menstrual period, mental disturbances decided at all times. After being under treatment of gynecologists for years, with negative results, oöphorectomy was advised, with the absolute alternative of death or the asylum. Marriage occurred soon afterward. That woman is to-day, with the exception of an anteversion, perfectly well as far as her uterine condition goes, has a normal menstruation, no mental trouble, and is well known in the South as a leader of society and a woman of brains.

CASE 4.—Has been reported by Dr. Lusk, as follows: The patient when I first saw her, some years ago, was a young woman of seventeen. She had suffered agonizing pains at monthly intervals, and was confined to the bed or couch for the greater part of the time. An examination revealed occlusion of the lower vagina. An opening was made and a large amount of retained blood and clots was removed from the upper vagina and uterus. The latter had been converted into a sac. For a long time thereafter the tubes remained thickened and tender. This was especially marked on the left side, to which the fundus of the uterus was drawn by peritoneal adhesions. Salpingotomy was plainly indicated, and I should have performed that operation had I obtained the patient's consent. Finally, however, she married. My advice in the matter was not asked. Last June the young woman called upon me. She had been married a year, and was seven months pregnant. The thing was inceivable, but it was a fact. I will simply add that labor was normal, and that this woman is to-day as perfect a specimen of physical health as I know.

The above is a brief *résumé* of the histories of the only four women that I have ever known that refused this operation. The time that has elapsed since it was advised varies from eighteen months to twelve years. I do not go into the details of the cases, as the only point I wish to make is, that in each case the operation was advised and urged by gynecologists both North and South, consequently they must have been convinced that these were cases demanding operative interference. Please compare the results obtained without operation with the results that would have followed the best operative success. Think at what cost cases two, three and four would have figured as successful cases in the statistics of the gentleman that wished to perform the operation—a possible sacrifice of marriage, and an absolute sacrifice of that greatest boon to all women, maternity. Cases three and four I have known most intimately since girlhood, and no language of mine can express the difference between what is and what might have been in these two cases.

In a practice of average size for twelve years I have had occasion to recommend the removal of the appendages once, excepting cases of ovarian tumors. That one proved to be a case of pyosalpinx. Several of my patients have drifted into other hands and had oöphorectomy performed, and as far as I can learn, have been disappointed in the results each time. The best men in this special line of work are doing this operation less and less each year. Their place is being amply filled by lesser lights, with smaller numbers of individual cases, but with a yearly aggregate that is terrible to contemplate. By what combination of circumstances could one man, to fame unknown, in a small interior city, and in a short space of time find one hundred and forty-four cases demanding abdominal section.

I will acknowledge that nearly all operators at present only operate for some pathological condition of the ovaries or tubes, in their conception. But one of our best pathologists does not agree with them, and he had usual opportunities of examining bottled ovaries.

Any one desiring to follow up the line of thought suggested by these cases will be interested in the following late articles:

"The Remote Results of the Removal of the Ovaries and Tubes," W. T. Lusk, *American Journal of Diseases of Women and Children*, Vol. 25, No. 11. "The Abuse of Oophorectomy in Diseases of the Nervous System," Allen McLain Hamilton, *N. Y. Medical Journal*, February 17th, 1893. "Abdominal Surgery and its Evolutions and Involutions," Joseph Eastman, *Journal American Medical Association*, March 11th, 1893.

